# Overview of the Potential Impacts of Climate Change on the Microbial Safety of the Dairy Industry

**DOI:** 10.3390/foods9121794

**Published:** 2020-12-03

**Authors:** Rodney J. Feliciano, Géraldine Boué, Jeanne-Marie Membré

**Affiliations:** Secalim UMR1014, INRAE, Oniris Chantrerie, CS 40706, CEDEX 3, 44307 Nantes, France; rodney.feliciano@oniris-nantes.fr (R.J.F.); geraldine.boue@oniris-nantes.fr (G.B.)

**Keywords:** food safety, microbial risk, milk, cow, temperature, weather changes

## Abstract

Climate change is expected to affect many different sectors across the food supply chain. The current review paper presents an overview of the effects of climate change on the microbial safety of the dairy supply chain and suggest potential mitigation strategies to limit the impact. Raw milk, the common raw material of dairy products, is vulnerable to climate change, influenced by changes in average temperature and amount of precipitation. This would induce changes in the microbial profile and heat stress in lactating cows, increasing susceptibility to microbial infection and higher levels of microbial contamination. Moreover, climate change affects the entire dairy supply chain and necessitates adaptation of all the current food safety management programs. In particular, the review of current prerequisite programs might be needed as well as revisiting the current microbial specifications of the receiving dairy products and the introduction of new pretreatments with stringent processing regimes. The effects on microbial changes during distribution and consumer handling also would need to be quantified through the use of predictive models. The development of Quantitative Microbial Risk Assessment (QMRA) models, considering the whole farm-to-fork chain to evaluate risk mitigation strategies, will be a key step to prioritize actions towards a climate change-resilient dairy industry.

## 1. Introduction

Climate change is one of the most pressing concerns that our world is facing today. It is attributed directly or indirectly to human activity that alters the composition of the global atmosphere and is in addition to natural climate variability observed over comparable time periods [1]. These changes associated with climate change include the increase in mean average seasonal temperature and increase in precipitation during winter or the wet season [2,3,4]. In addition, climate change also encompasses the alteration of ocean properties, such as surface temperature, acidification and lower levels of dissolved oxygen, due to the higher levels of CO_2_ and greenhouse gases in the atmosphere.

In this context, several researchers have already sounded the alarm on the possible effects of climate change on the supply of food products. Climate change is also projected to affect the physicochemical, sensory properties and safety of food products [5,6,7,8,9]. Although the full effects of climate change is yet to be seen, research has already shown the vulnerability of raw food products against the effects of climate during different seasonal and climatic shifts [10]. The effects of climate change in the food supply chain were validated by the observed reduction in the yield of major food crops such as rice, maize, apricots, peach and cauliflower [11,12,13,14,15], seafood products [16] and meat products [17]. On the contrary, in some exceptional situations, the middle and higher latitude regions might provide positive impacts [2,15,18].

Safe food, as defined by the European Commission, is a food that is not injurious to health and fit for human consumption [19]. Considering that definition, the effects of climate change on the microbial changes of dairy products presented in this review encompass both the safety related to foodborne pathogens and spoilage microorganisms. Climate change effects, such as relative humidity, daily temperatures and precipitation, were shown to influence the survival of pathogens in food crops, particularly green leafy vegetables, and in the adjacent environment [13,20,21,22,23,24,25]. On the other hand, wind speed and ultraviolet radiation were both found to decrease the level of *Enterobacteriaceae* on fresh produce [10,25]. In turn, extreme weather events, such as flooding, were shown to increase the prevalence of *Escherichia coli* and *Salmonella* spp. [26,27]. In addition, rain splashing and storm water runoffs were also shown to facilitate the dispersal of microbial contamination from soil to crops [22,28].

The dairy industry is a particular sector that is vulnerable to climate change given the sensitivity of its parts to existing weather conditions [29]. A foretaste of these future effects can already be seen with news reports showing the decline in raw milk yield due to the increase in average temperatures on farms [30]. As such, the economic implication of this decline in milk yield is expected to increase by the end of the century [31,32]. Nevertheless, dairy products are expected to have a continued sustained demand for its wide-ranging products because of its role in human nutrition and as among the most traded goods in the world [33,34]. However, given the projected intensification of the brute effects of climate change, the dairy-producing countries located in the northern hemisphere are predicted to see an increase in productivity due to warmer climate brought about by climate change [2]. Therefore, the objective of this paper is to present an overview of the effects of climate change on the microbial food safety of the dairy supply chain and suggest mitigation strategies to limit the impact of climate change effects on the dairy sector.

## 2. The Status Quo in the Dairy Supply Chain: From Food Safety Management to Microbial Hazards

### 2.1. Food Safety Management: An Overview

The microbial food safety of dairy products is currently met by dairy manufacturers through adoption of food safety management systems and food safety metrics. These food safety management systems are now well established; they are built upon working prerequisite programs [35], including Good Hygiene Practices (GHP), Good Agricultural Practice (GAP) and Good Manufacturing Practices (GMP), as well as Hazard Analysis and Critical Control Point (HACCP) and other guidelines that aim to address issues in food safety and delivering quality products [35,36,37]. In the dairy supply chain, the implementation of these prerequisite programs are more suitable at the earlier parts of the farming and milking practices than the use of food safety programs [38,39,40]. Complementary to these are metrics such as the Appropriate Level Of Protection (ALOP) and Food Safety Objectives (FSO) that were developed within a risk-based food safety management concept in order to be applied in connection to prerequisite programs and HACCP plans [41]. At the industrial level, they are translated into Performance Objectives (POs), Performance Criterion (PC), Process Criteria (PrC) and Product Criteria (PdC) [35,41,42,43]. Quantitative Microbial Risk Assessment (QMRA) [44,45,46] was also developed to quantify the public human health risks associated with certain dairy products’ consumption [47,48]. Combining the application of QMRA with the establishment of food safety and prerequisite programs in dairy processing facilities are the cornerstones in achieving food safety [49,50].

### 2.2. The Dairy Supply Chain and Introduction to Different Dairy End Products

**Figure 1:**
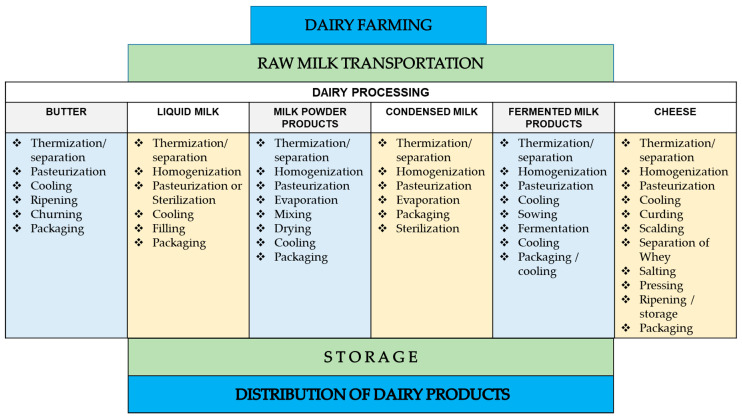
Scheme of the dairy supply chain from raw milk to the distribution of dairy products with unit operations [54,56,57].

The food supply chain is a series of multi-system processes that a food product undergoes until its consumption. This encompasses the farm to fork continuum and acknowledges the fact that the practices involved during farming, transportation, processing, storage of food and consumer practices have individual and compounding impacts on the safety of food prior to consumption [51,52]. This approach has been applied in understanding the complexity of food systems, such as the dairy supply chain [53]. The dairy supply chain is multifaceted with different end products that are derived from a common raw material, which is raw milk [54]. These products are produced through the succeeding food processing operations emanating from raw milk (Figure 1).

The microbial food safety of dairy products was previously presented by the International Committee on the Microbial Specification of Foods (ICMSF). The microbial ecology of raw milk depend on the contamination of the udder of the cow (interior and exterior surfaces), milking equipment, environment (air and water sources) and persons handling the milk [49,55]. These factors will impact the microbial load and diversity that must be reduced during the subsequent unit operations. Dairy products undergo four common stages in the dairy supply chain, namely, dairy farming, raw milk transportation, dairy processing and distribution of processed dairy products [9,53]. With these, the significance of the farm-to-fork continuum can be seen, where the impacts of food safety processes during the early parts of the dairy supply chain can be compounded and multiplied at the end prior to consumption.

### 2.3. The Microbial Hazards in the Dairy Supply Chain and its Current Controls

Food safety hazards in the dairy supply chain include pathogenic and spoilage microorganisms as well as mycotoxins produced by fungi. For raw milk, some of the microbial hazards, such as *Mycobacterium* and *Brucella*, are controlled due to the widespread control at the veterinary level and adoption of good agricultural practices (GAP) [52,55]. These microbes, along with other hazards associated with the liquid dairy milk products, are currently controlled through the stringency of the heat treatments applied. However, this is not the case for chemical hazards of biological origin, such as mycotoxins and staphylococcal toxins, which are thermal processing stable [9,58,59]. Pathogenic microorganisms introduced at the initial farming phase are usually reduced by the severity of the applied thermal processing conditions and duration of the treatment, such as with ultra-high-temperature processed milk (UHT) (135–150 °C thermal treatment, 3–5 s duration at different cycles), high-temperature, short-time processed milk (71–78 °C, 15 s) and pasteurized milk (62–65 °C for 30–32 min) [49].

The efficiency of these heat treatments is not without difficulties as spore forming microorganisms are able to survive pasteurization and low-temperature, short-time processing, while thermoduric microorganisms, such as *B. sporothermourans*, are able to survive the UHT processing of milk. In addition to these are the occasional occurrence of cross contamination in line, where the manufacturing equipment has been linked to biofilm formation. An example of these are the harbouring of *Streptococcus thermophilus*, *Bacillus lichenformis*, *Geobacillus stearothermophilus*, *B. sporothermodurans* and other thermophiles in heat exchangers [60,61]. Another form of cross-contamination relates to the recovery mechanisms in a manufacturing line. An example of such an event has been reported, where *Cronobacter* spp. contamination in powdered infant milk formula was linked to industrial air filters. During the spray drying operations, some of the powder is carried out together with the drying air [62]. The powder is recovered by filtering the air–powder mixture using an air filter. This is prone to harbour *Cronobacter* spp. and were the source of contamination of the dairy end-product. A similar example is the staphylococcal toxin contamination of skimmed milk powder due to the temperature abuse of the recovered milk, which caused a foodborne disease outbreak [59]. In this setup, residual milk concentrates are recovered with water and mixed with raw milk that, if not stored in proper conditions, will support the growth of *Staphylococcus aureus* and its toxin production.

Post-thermal processing and the related unit operations associated with it remain a crucial challenge where the reintroduction of spoilage microorganisms and pathogenic microorganisms occurs. Among these are spoilage microorganisms, which includes *Geobacillus stearothermphilus*, *Micrococcus*, *Bacillus cereus*, *Anoxybacillus flavithermus*, *Pseudomonas fluorescens*, *Enterobacter faecium* and *E. faecalis*, and occasionally pathogenic microorganisms such as *Salmonella* and *Listeria monocytogenes* [55,63,64,65,66]. These microbial hazards are currently controlled through the design of the equipment, environmental hygiene and routine testing [49,67].

Fermented milk products, on the other hand, are generally stable due to their acidification and production of biopreservatives (lactic acid, bacteriocin, etc.) by *Lactobacillus*, *Bifidobacterium*, *Lactococcus* and *Pediococcus*, among others [68,69]. Spoilage of fermented milks due to the overgrowth of yeasts and low acid fungi contamination were linked to initial slow growth of the lactic acid culture and a decrease in lactic cultures due to the occurrence of phage contamination [55,70]. Pathogenic microorganisms found in fermented milk are associated with post-processing contamination or due to an improper pasteurization process or use of contaminated raw milk [55]. As such, food safety controls, such as thermal treatments prior to the fermentation step, are still recommended to be applied [71].

Cheese microbial ecology is complex; its dynamics have been previously elaborated by research monitoring the changes from its surface to inside structure as the cheese was ripened through time [72,73,74,75,76]. Common pathogenic microorganisms associated with cheese are introduced with the use of raw milk, failure in the process of cheese making and possible environmental contamination during the ripening stage [55]. In turn, the hazards associated with mycotoxin production in cheese have been associated with contamination during the initial parts of the dairy supply chain during farm practices and possible mould growth during the ripening stage [77,78,79]. As such, it is recommended that control over the processing schedules and good hygiene practices be applied throughout the dairy supply chain [49,55].

In light of these existing challenges with the microbial food safety of dairy products, a new threat in the dairy supply chain is foreseen—climate change. Several researchers have sounded the alarm on the additional impact that climate change might impose on the different parts of the dairy supply chain, particularly on the microbial contamination of raw milk [80,81,82,83,84].

## 3. Effects of Climate Change on Raw Milk and the Dairy Supply Chain

### 3.1. Effects of Climate and Seasons on the Microbial Ecology of Raw Milk

**Figure 2:**
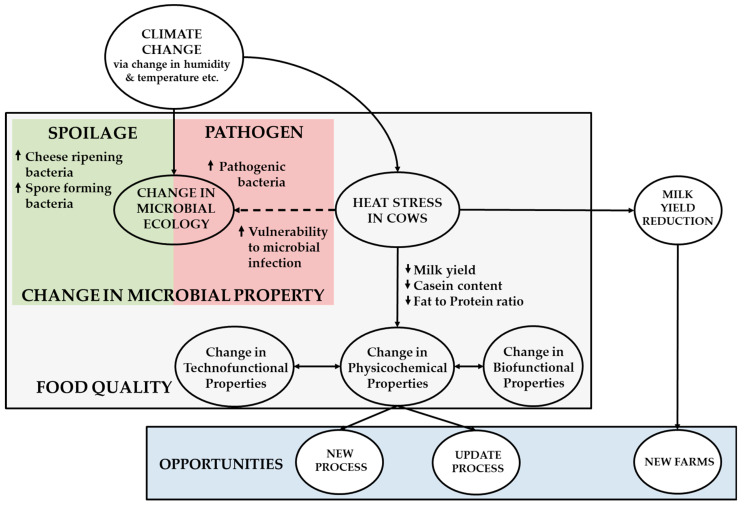
The effects of climate change on raw milk.

Climate change is expected to influence the microbial profile of dairy products through the direct impact of climate variables and seasons on the microbial ecology of raw milk (Figure 2) [5,9]. These climate variables may include changes in average temperature, relative humidity, average precipitation and sunlight exposure, among others, which are also associated with different seasons [24,85]. Changes in climate variables can lead to the rise of pathogenic or spoilage microorganisms in raw milk. On the other hand, the indirect impact of climate change on the microbial profile of raw milk will occur through the induction of heat stress in lactating cows, which influences their susceptibility to pathogenic microorganisms, changes their microflora and, ultimately, the concentration and types of microorganisms in raw milk. In addition, associated with the heat stress in lactating cows are the reduction in milk yield and changes in the physicochemical properties of the raw milk obtained from these cows.

The projected rise in global mean temperature is expected to enable the faster growth of microbes and also changes in the microbial ecology of the raw and processed milk products [9,86]. These changes can have two sides: the increase in microbial load is from foodborne pathogens and/or food spoilage microorganisms. Several researchers have already shown that seasons and climate influence the microbial ecology and diversity of raw milk [87,88,89,90,91].

Among the four seasons, it was found that the summer season had the most diverse microorganisms followed by spring and autumn, while the least microbiologically diverse milks were obtained during the winter periods [86,90]. In terms of the microorganisms present in raw milk, bacteria belonging to *Paenibacillus* and *Bacillus* were predominantly found during summer in Australia [91]. In Israel, the prevalence of *Bacillus* and other bacteria belonging to the *Gammaproteobacteria*, *Actinobacteria* and *Flavobacteria* was reported in milk during summer [87]. Similar results were reported from milk samples obtained in China during the summer, with the bacteria found in milk belonging to *Bacillus*, *Lactobacillus*, *Bifidobacterium* and *Acinetobacter* [86].

During spring, it was found that the predominant bacteria in milk include *Corynebacterium*, *Aerococcacea*, *Knoellia*, *Enhydorbacter* and *Acinetobacter* [90]. Similarly, it was reported that spring and autumn isolates from Australia are similarly composed of *Serratia*, *Hafnia*, *Klebsiella*, *Acinetobacter* and *Pseudoalteromonas* [91]. While, in Israel it was reported that the microbial ecology of raw milk for spring and winter are quite similar, with *Gammaproteobacteria* (includes *Pseudomonas* and *Acinetobacter*), *Bacilli*, *Enterococcus*, *Leuconostoc*, *Staphylococcus*, *Lactobacillus*, *Actinobacteria* and *Flavobacteria* [87]. Winter milk samples collected in Normandy, France, were reported to be composed of Gram-negative and presumptive *Lactococcus* [88]. Moreover, they found that *Lactococcus*, *Lactobacillus*, *Leuconostoc* and yeasts were correlated with the winter and spring samples. *Pseudomonas* and *Lactobacillus* also were found in milk from Norway [92]. While others found *Bacteriodetes*, *Staphylococcus*, *Fibrobacter*, *Acidobacteriales* and *Coxiella* [90]. On the other hand, it was reported that *Pseudomonas*, *Acinetobacter*, *Psychrobacter* and *Bacillus* were higher during the winter season [91]. Together with *Propionibacterium* and *Flavobacterium*, *Pseudomonas* was also prevalent for milk samples obtained during winter [86].

These previously mentioned studies have shown that summer contained higher levels of microorganisms and the most reported ones were spore-forming microorganisms, such as *Bacillus* spp. The microorganisms commonly found by researchers during winter were lactic acid bacteria, *Pseudomonas* spp. and other psychrophiles. On the other hand, similarities between seasons were observed by some researchers, namely between autumn and spring and winter and spring. Occurrences of these similarities might be related with the similar weather conditions between these two succeeding periods. The relevant insight is clear, in that the seasonal patterns of microorganisms are linked to the weather conditions affecting the microbial ecology of raw milk. However, further study is still needed in order to determine clearly what group of microorganisms will be favourably influenced by the climate change-driven weather conditions and extreme events, such as flooding and dry spells. It can be inferred that those predominant during summer might be able to persist during elevated temperatures and heatwaves, while those commonly found during autumn and winter will be able to grow during wet seasons. Notwithstanding the fact that variability in latitudes and current weather conditions already influence the microbiology of raw milk from these areas. The influence of climate change-driven changes in weather will add to the need of a localized understanding of the situation, which is still needed for adopting mitigation strategies rather than adopting a global approach.

### 3.2. Heat Stress in Cows: Influence on Microbial and Physicochemical Properties of Raw Milk

Climate change is expected to increase the average temperatures and occurrences of extreme weather conditions, such as droughts [2], which might impact the dairy industry by altering the health of lactating cows, ultimately impacting the quality of the raw milk (Figure 2). Indeed, the impact of hotter conditions can influence the induction of heat stress in cows. Heat stress can be defined as the effect of hot, humid conditions on the normal resting state of a cow, resulting in disturbances of its normal productive or physiological conditions [93]. These can include the impairment of immune functions, induction of oxidative stress and decrease in eating habits in lactating cows [94]. Associated with these changes in physiological conditions are their increased susceptibility to infections and vulnerability to mastitis [95,96]. On the other hand, given that the climate change-driven emergence of new pathogens and vector-borne diseases is a possibility in the future, these might add to the challenge of maintaining cow health under climate change scenarios [9,97]. Ultimately, these alterations on the health status of cows can also bring about changes in the raw milk’s microbiology, a reduction in raw milk yield and alteration of the raw milk’s physicochemical properties [98].

Mastitis in lactating cows is commonly caused by bacterial infections, such as those from *S. aureus*, *E. coli*, *Streptococcus* spp. (*S. agalactiae*, *S. dysgalactiae* and *S. uberis*) and, although seldom, *Listeria* spp. (*L. monocytogenes*, *L. innocua* and *L. ivanovii*) [90,95,98]. Due to the use of antibiotics, resistant forms of these microorganisms were also isolated in lactating cows and milk [95]. Ultimately, these microorganisms are passed into the raw milk, including those obtained from cows that are not presenting symptoms of mastitis, as in the case of a subclinical form of mastitis [99]. The future effects of climate change on mastitis can be gleaned from research showing the influence of climate conditions across different seasons on mastitis in lactating cows. It has been shown that the occurrences of clinical mastitis in Holstein cows in Italy varied per season due to reduced heat stress and lower temperature heat index (THI) [100]. The highest clinical mastitis incidence rates were observed during the summer season, particularly for the months of July and June, with incidence rates of 3.62 and 3.16 at a THI of 79.2 and 75.6. The lowest incidence of mastitis was observed for winter (incidence rate of 2.58 at a THI of 58.9) followed by autumn (incidence rate of 2.54 at a THI of 70.5). On the other hand, researchers have shown that Irish herds of cows from the UK were found to contain higher levels of somatic cell counts during the spring and summer periods, meaning that the cows in the herd during these seasons have mastitis [101]. Similarly, it was found that the prevalence of somatic cell counts in heifers during summer (23.8% of cows) were the highest among the four seasons in Switzerland [102]. Associated with mastitis are other changes in the physicochemical and technofunctional properties of the raw milk and the products derived from it [103,104].

Heat stress in cows was reported to have a negative influence on milk yield and physicochemical properties of raw milk [93,105,106,107]. The negative influence of heat stress results in a reduction in the milk yield obtained in cows during lactation. It was reported a 0.41 kg decrease in milk yield per cow per unit in the temperature heat index (THI) after reaching the threshold of 69 THI units [105]. A decrease of 27.6% (9.6 kg) in milk yield under experimental conditions was reported [107]. While a positive correlation between the temperature heat index and milk yield was reported, where an increase of 1 unit in the temperature heat index resulted in a decrease of around 0.22 to 0.52 kg of milk yield per day per cow in Brazilian Holstein cows [106].

The associated effects of heat stress on the physicochemical properties of raw milk include the decrease in milk protein, lower casein content, lower level of fat and changes in the fatty acid profile [80,81,82,83,84]. An example of the implication of these changes is the decline in protein content, particularly the reduction casein. This reduction will make it difficult to form a cheese block, which is a technofunctional property of casein in milk [80]. The economic impact of this is through the reduction of cheese yield from milk. Moreover, these reductions in casein and other milk proteins will also impact the bio-functional properties of milk, such as its antihypertensive and hypolipidemic activities [108,109,110]. Another biofunctional property of casein in milk that is expected to decline due to climate change is its use as excipient food by aiding the absorption of bioactive compounds in foods during digestion [111,112].

In summary, the effects of climate change on raw milk not only include changes in the microbial profile, such as the increase in pathogens and spoilage microorganisms, but also changes in the physicochemical, biofunctional and technofunctional properties of the raw milk and products derived from it. Changes in these properties were brought about by induction of heat stress as a direct consequence of heat stress in lactating cows. These changes in the properties in raw milk and cow physiology are expected to impact the status quo in the dairy supply chain and new opportunities might be explored in light of these expected effects.

### 3.3. Climate Change Effects along the Dairy Supply Chain

Evidence of the possible effects of climate change along the dairy supply chain was shown by some researchers. It has shown that the microbial diversity of raw milk from different suppliers were influenced by location and season [113]. In turn, changes in climate during raw milk handling and transportation, such as higher average temperature and extreme weather conditions (e.g., storms and high precipitation changes) might be relevant as it may influence the growth of both spoilage and pathogenic microorganisms in these stages [113,114].

Impacts of climate change on dairy farming practices and processing can be gleaned from the insights provided by previous studies, where the influence of initial microbial load and microbial diversity on the subsequent steps of the dairy processing chain were seen [115,116]. Another relevant effect of climate change is on the dairy farm and food processing environment itself, which might be affected by extreme weather conditions such as drought and flooding due to precipitation, as reported for some dairy farms [117,118,119]. The temperature conditions at the distribution locations have been found to significantly influence the shelf-life and spoilage risk of dairy products, such as evaporated milk, by enabling the growth of spore forming bacteria [64].

The evidence has shown that future changes in weather conditions will not only impact the microbial ecology of raw milk to be used for processing but also the different parts of the dairy supply chain; this given the possible changes in the hazards and concentration levels due to the respective localized climate changes in the different parts of the food supply chain. Food safety management and farms should review their susceptibility to these effects and whether there is a need to change current practices or even the location of their facilities, where the effects of climate change are expected to be fully felt. Changes in food safety management at the farm and factory level might be needed dependent upon the projected changes in climate pattern and the vulnerability of the different parts of the dairy supply chain.

## 4. Towards a Climate Change-Resilient Dairy Supply Chain: Development of Climate Responsive Mitigation Strategies in Food Safety Management

### 4.1. Developing QMRA Models Integrating Climate Change Effects

**Figure 3:**
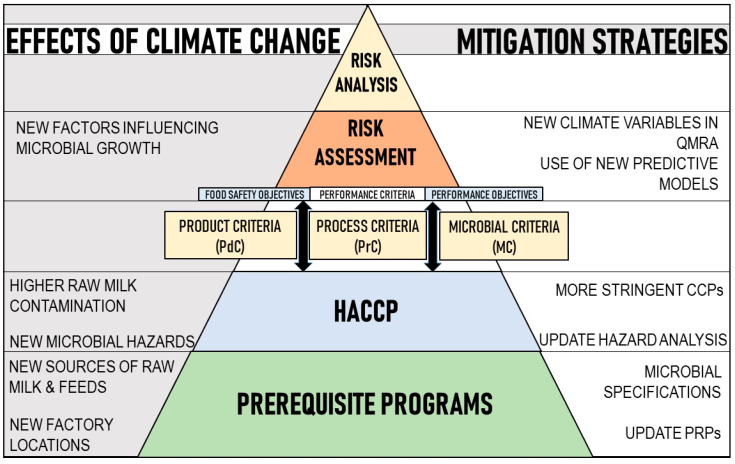
Food safety management approach to climate change: from the effects of climate change to mitigation strategies (adapted from [35,41]).

Presented in the previous sections was the evidence of the influence that climate exerts on the dairy supply chain from the production of raw milk to the shelf-life of food products. The roles of the current food safety management and food safety controls in minimizing microbial hazards and achieving food safety were underscored. With this in mind, mitigation strategies can be approached in two ways: first through changes in food safety management, and second through adaptation of a climate-responsive mitigation strategy in which QMRA will have a significant role (Figure 3).

Climate events, such as an increase in the daily temperature and amount of precipitation, were shown to influence the microbes in raw milk while the heat temperature index was able to induce heat stress in lactating cows. Developing quantitative risk assessment models, which are adapted or responsive to climate change, accounting of these different climate events in the QMRA model is necessary. Structural changes in QMRA have been proposed as a preparation against the possible effects of climate change [120,121,122]. However, ways on how to incorporate climate change effects in quantitative risk assessments for dairy products are yet to be done.

Several approaches on how climate change effects can be incorporated as a variable in a QMRA can be found in research involved in the production of safe green leafy vegetables [24,123,124,125]. From these research studies, three methods on how climate change effects can be inputted in a QMRA model were shown. First, through direct incorporation of climate data with a probability distribution [126,127,128]. Second, through the dimension reduction of the available climate data and selection of the most significant environmental variable to be incorporated into the QMRA model [24,124]. Third, the development of Bayesian networks model to quantify the influence of climate data on microbial contamination [129,130].

The direct incorporation of climate variables has been shown where precipitation events and sunny days were inputted in the models with their probability distributions [126]. The influence of these two climatic events on the increase in the daily *E. coli* concentration were then computed. On the other hand, seasons have been incorporated as a variable in performing the exposure assessment part of the QMRA [128]. A converse way of taking into account climate change in a QMRA is through the incorporation of the effects of climate change (e.g., *E. coli* levels due to climate conditions) rather than the climatic events itself (e.g., wind speed and UV radiation level) [127,131]. A QMRA was performed to determine the risk in the consumption of baby spinach and rocket lettuce subject to the effects of handling conditions (*E.coli* inoculation in the fresh produce via irrigation water, *E.coli* inactivation via temperature and sunlight exposure in simulated conditions, rinsing of vegetables) [131]. The inactivation of *E. coli* was performed in a climate chamber and was associated with temperature and light intensity in the produce. Performing the QMRA, the authors used different scenarios where the levels of *E. coli* after the inoculation event were influenced by the temperature and light intensity. A similar approach was followed in another study where the days that the produce remains in the field after the contamination event was an input in the QMRA model [127]. However, this attribution of the effects of climate change rather the climate event itself must be done with caution given that a clear causal relationship between the climate event and the effects does not exist.

Dimension reduction techniques on the climate data were shown to be effective in quantifying how temperature, precipitation and wind speed data influence the prevalence and level of *E. coli* contamination in fresh produce [124]. Logistic regression (univariate and multivariate methods) and classification tree modelling were performed on the collected climate data to understand the influence these exerts on the microbial contamination of fresh produce and the most significant variable was selected. In turn, principal component analysis has been used for dimension reduction on relative humidity, rainfall and radiation, to understand the influence of these environmental factors on the contamination of lettuce by *Pseudomonas* spp., coliforms and mesophilic aerobic bacteria [132]. The relationship between the selected variables were then characterized mathematically using linear regression equations. These dimension reduction approaches, if applied to climate data that are to be incorporated into the QMRA models, can help risk assessors quantify the effect of climate events.

Incorporating climate events in secondary predictive models can also be explored by performing simulation studies investigating the influence of climate factors. An example of this is the milk model developed for the growth of *L. monocytogenes* in pasteurized whole milk [133]. In this study they have determined the growth of the pathogen at different temperature conditions and used these data in the development of the model. This approach of model building can be used where the different temperature conditions to be tested are the projected temperature effects of climate change. Another approach is through the use of design of experiments where some of the factors to be incorporated are climate variables or storage conditions. Simulation studies will then be performed and assessed through microbiological challenge testing or shelf-life studies. Similarly, the data obtained through these simulation studies can also be processed using Artificial Neural Networks and Bayesian networks in quantification. Ultimately, secondary models to be obtained from these can be used in the appropriate modules of a QMRA model.

### 4.2. Food Safety Management Options as Mitigation Strategies in the Dairy Supply Chain

#### 4.2.1. Dairy Farming Stage

The dairy farming stage considered encompasses agricultural farming practices, milking practices, raw milk pooling and hold-on in pooling tanks prior to transportation to dairy processors [134]. The effects of climate change on raw milk, as previously mentioned, are changes in the microbial ecology and load of the raw milk and induction of heat stress in cows. Thus, the mitigation strategy for the former is through introduction of hygienic procedures or pretreatment steps and more stringent veterinary health maintenance. While, for climate change-driven heat stress, several researchers have suggested strategies that can reduce these at the farm level [97,135]. Incorporating these strategies will be dealt with at the first level of food safety management through a review of the current prerequisite programs, GAP, GHP and even Good Veterinary Practice (Figure 3).

For the first possible effect of climate change, a concrete example of the proposed mitigation strategy is by implementing or revisiting the procedures for cleaning the udder of the cow, milking equipment and surfaces prior to milking [136,137]. If already being done, the use of alternative cleaning technologies, such as use of probiotics or other disinfectants, might be considered [138,139]. More stringent cow health maintenance using vaccination and probiotic treatment also might be considered given their increased susceptibility to bacterial infection (e.g., Shiga toxin-producing *Escherichia coli*) and shedding due to climate change [140,141]. Establishment of these new cleaning procedures and maintaining animal health aims to contribute in meeting the microbiological specifications of a dairy manufacturer and cushioning the possible effects of climate change. Incorporating these changes will necessitate the updating of current GAP and GHP implemented at the farm level.

A converse approach to these can be through the prevention of heat stress in cows and interventions in terms of veterinary practice that can help them adapt in environmental conditions. These include changes in feeding regimes, adaptation of cooling infrastructures and consideration of the breed characteristics of the cow [93,97,135]. For the feeding regimes, given the changes in the feeding behaviour of the cows during hot conditions, adaptation of feed and feeding or drinking times should be adapted in order to meet their dietary requirements. An example that was brought up is the feeding times can be reinforced at times of the day where the temperatures are lower and cows are more ready to eat [97]. Incorporation of cooling infrastructure, such as misters, sprinklers, fans, roofing and shade, might need to be employed in the future [97,142]; also, a selection of cow breeds with certain genetic profiles that allow them to have higher resistance to heat stress might also be a form of mitigation strategies in the future [93,106,143].

New sources of raw materials for cow feed and raw milk sources are options that can be considered during occurrences of extreme weather conditions, such as droughts or floods [9]. However, these options might come at a cost given the possible changes in the microbial profile of these raw materials [88]. Nevertheless, food manufacturers can address these in their food safety management systems by updating their current prerequisite programs and their microbial specifications for audited suppliers.

#### 4.2.2. Transportation of Raw Milk

Milk transport includes the condition of transportation until the raw bulk milk enters the processing line up to component separation prior to thermal processing. An option recommended in this part of the dairy supply chain is the establishment of assurance systems and proactive monitoring systems of the transport conditions [120,144]. One of the recommendations proposed is to establish proactive monitoring systems that will monitor the extreme climatic events with a forecast of its impacts towards the safety of food products and the food supply chain [144]. These tools will be useful in coming up with a response subject to the possible effects on meeting the microbial specification of the dairy processors. Establishment and inclusion of these monitoring systems in prerequisite programs must be validated and verified from time to time, which means there will be a need for food manufacturers to gather real-time data that may be used not only in present monitoring programs but also for the future progression of climate change effects.

#### 4.2.3. Dairy Processing and Post Processing

The dairy processing stage for the different dairy products shows the different unit operations until its packaging prior to distribution. These respective unit operations are usually operated in the same facility. As such, the possible mitigation strategies might be through changes in the current food safety programs and prerequisite programs to limit the projected effects of climate change (Figure 3). Mitigation strategies proposed are the establishment of stringent processing regimes and introduction of pre-treatment processes.

The option to implement stringent processing regimes may come as an increase in the required log reduction value, after expert reviews, because these values are already very stringent (for pasteurized milk a 5 log reduction of vegetative bacteria; 9 log reduction of thermophilic spores for commercially sterile milk) [55,145]. Another alternative could be the introduction of non-thermal processing technologies as a hybrid with thermal treatments in order to decrease the microbial impact incurred by climate change effects. However, application of these non-thermal processing schedules must take into account the initial inactivation resistance of the microorganisms to these technologies, which might result in the overestimation of its inactivation efficiency [146].

The post-processing part is a significant source of recontamination in milk and other dairy products mentioned [67,78,147]. Currently, food safety programs and routine environmental testing is used to monitor the possible sources of post-processing contamination. Revisiting the currently implemented systems might be needed depending on the projected vulnerability of the facility to the effects of climate change and extreme weather events. As such, it was recommended to implement effective food safety programs to avoid inoculation of moulds producing mycotoxin during the cheese ripening stage [45].

The post-processing stage was also identified to be a significant recontamination point impacting the microbial food safety of different dairy products [148,149]. In a study on a UHT processing line, biofilm attachment and air velocity conditions during the post-processing impact sterility failure rates in packaged milk products [148]. Similarly, it was shown that the probability event of *Listeria monocytogenes* recontamination is a critical point in the manufacturing of soft cheese [149].

#### 4.2.4. Distribution and Consumption of Dairy Products

The last part of the dairy supply chain presented is the distribution of products from the food manufacturing environment. As such, with the projected climate change effects, the optimum conditions during the distribution until retailing might be harder to maintain. Climate conditions and temperature during the distribution, retailing and consumption of the shelf-life of dairy products were shown to impact the microbial food safety of evaporated milk and pasteurized milk [64,150]. This is true for the presented dairy products, where most of the current storage conditions require a low temperature during storage. As such, new microbial risks might be associated during this part of the dairy supply chain with an increase in storage temperatures. Therefore, as a mitigation strategy it is underscored that the temperature control during transport and retailing might be revisited due to higher average temperatures and occurrence of extreme weather conditions that might occur due to climate change. In conjunction with this the use of climate event early warning systems have been proposed to provide risk managers real-time data for improving the responsiveness to these climate events [144]. In addition, data gathered during climate monitoring can be used as inputs in a climate change-responsive QMRA model. Through this approach dairy manufacturers will be able to estimate the number of product units that will fail given the effects of climate change [64,151].

Several studies have also pointed out the importance of consumer practices and refrigerators in impacting the shelf-life of dairy products and used these data in estimating the shelf-life of dairy products [152,153]. Incorporation of these data from the consumer level and the possible influence on these by climate factors is worthy to look at given its influence on the effectiveness of the refrigeration of foods and on consumer behaviour towards foods in their homes during extreme hot weather. Incorporation of these data in a separate module dedicated to consumer handling in a QMRA model, in order to better estimate the shelf-life of dairy products during hot weather or extreme weather events, is suggested. The implications of these might necessitate changes in product formulation, changes along the manufacturing of foods or changes in the shelf-life of the product in order to ensure that the dairy products are appropriate to consume given the consumer’s possible handling of the product.

## 5. Conclusions

Climate change effects, through increases in average temperatures, increased occurrences of heat waves or increases in rain and wet seasons, might require adaptation changes of practices all along the dairy supply chain in terms of microbial food safety. Indeed, it was shown that all stages from farm to consumption are vulnerable to climate change: raw milk production and transportation, dairy product processing and distribution and storage of the end-products. Raw milk is a particularly at-risk product, to which climate change is expected to influence its microbial profile and indirectly through heat-stressed cows, making them vulnerable to microbial infection. Adaptation can be through changes in farm infrastructure (e.g., addition of ventilation or misting during summer or relocation of farms that are located in flood-prone areas). Moreover, impact on the production quality of milk in terms of microbiology and quantity of milk can also occur. For the former, more studies are still needed to further see the current impact of climate change over the last century and to model projections as we enter the climate change era. However, a foretaste of these can be seen through the microbiology of raw milk obtained during different seasons. On the other hand, the production quantity and quality are projected to decline, as shown by the previously mentioned studies. Predicting changes in the future is still needed at localized levels, where the specific impacts of climate change is expected.

The dairy manufacturing industries will have to review their current food safety management pre-requisite programs and HACCP considering the impacts of climate change. Together with these, the use of quantitative tools, such as the QMRA and predictive microbiology embedded into risk-based food safety management, will help in limiting the potential additional risk due to climate change. The role of food safety management in delivering safe food is crucial, as previously emphasized by food safety experts [41,43,49]. Adoption of the proposed mitigation strategies and development of new QMRA models will be a step towards a climate change-resilient dairy manufacturing industry.
